# DMF Activates *NRF2* to Inhibit the Pro-Invasion Ability of TAMs in Breast Cancer

**DOI:** 10.3389/fonc.2021.706448

**Published:** 2021-08-12

**Authors:** Ying Li, Yaxu Jia, Yurong Xu, Kan Li

**Affiliations:** ^1^Department of Clinical Laboratory, Affiliated Zhongda Hospital of Southeast University, Nanjing, China; ^2^Department of Epidemiology, School of Public Health of Suzhou University, Suzhou, China

**Keywords:** TAMs, DMF, *NRF2*, anti-cancer drug, breast cancer

## Abstract

Tumor-associated macrophages (TAMs) account for more than 50% of the cells in the tumor immune microenvironment of patients with breast cancer. A high TAM density is associated with a poor clinical prognosis. Targeting TAMs is a promising therapeutic strategy because they promote tumor growth, development, and metastasis. In this study, we found that dimethyl formamide (DMF) significantly inhibited the tumor invasion-promoting ability of TAMs in the co-culture system and further showed that DMF functioned by reducing reactive oxygen species (ROS) production in TAMs. The orthotopic 4T1 cell inoculation model and the spontaneous mouse mammary tumor virus-polyoma middle tumor-antigen tumor model were used to evaluate the antitumor effect of DMF. The results showed that DMF significantly inhibited tumor metastasis and increased T-cell infiltration into the tumor microenvironment. Mechanistically, *NRF2* activation was necessary for DMF to exert its function, and DMF can play a role in breast cancer as an anticancer drug targeting TAMs.

## Introduction

Breast cancer is a malignant tumor with a high incidence among women worldwide, presenting as a heterogeneous disease. According to the expression differences of estrogen receptor (ER), progesterone receptor (PR), and human epidermal growth factor receptor 2 (HER2), breast cancer can be divided into four molecular subtypes. That is, luminal A (ER^+^/PR^+^HER2^-^), luminal B (ER^+^/PR^+^HER2^+^ or ER^+^/PR^+^ Ki67 > 14%), HER2 (ER^-^PR^-^HER2^+^), and triple-negative breast cancer (TNBC, ER-PR-HER2-). There are prominent differences in the incidence, treatment response, and risk factors of the different subtypes (1). According to the treatment guidelines, endocrine intervention, chemotherapy, and other drugs are used as the first-line treatment for patients with breast cancer in China. Selective estrogen receptor modulators, selective estrogen receptor downregulation, aromatase inhibitors, docetaxel, or synergistic drug regimens are mainly used for treatment. These treatment strategies are the most important elements in the comprehensive treatment of patients with breast cancer as they have remarkable curative effects, greatly improving the clinical evaluation endpoints such as overall survival (OS) and progression-free survival (PFS). However, because the drug resistance rate reaches 30%–40%, the recurrence and metastasis of drug-resistant related tumors remain to be bottlenecks in the clinical treatment of various subtypes of breast cancer (2). Therefore, it is urgent to formulate new, innovative, and effective methods to deal with this.

Numerous studies have shown that the tumor microenvironment has obvious malignant and non-malignant cell components (3). Tumor-associated macrophages (TAMs) account for up to 50% of the tumor mass (3, 4). High TAM density is associated with a poor clinical prognosis in patients with solid tumors, including breast cancer, prostate cancer, cervical cancer, and ovarian cancer (5). TAMs are the key cells connecting inflammation and tumors, which can directly promote the occurrence, development, and metastasis of tumors by releasing various inflammatory factors, growth factors, and matrix proteases, or by indirectly promoting tumor progression by mediating tumor angiogenesis and immunosuppression. TAMs have become an important target for cancer treatment. Conditional *M-CSF* knockout of mammary epithelial cells leads to TAM deletion, which then leads to a delay in tumor progression and an inhibition of lung metastasis in a mouse model of T-type oncoprotein (PyMT) in ER mammary epithelial cells (6, 7). These findings suggest that the TAM phenotype and function are key factors in promoting tumor growth.

Fumaric acid, also known as dimethyl formamide (DMF), is an important intermediate metabolite in energy metabolism and a common industrial raw material. DMF and its metabolite, monomethylfumarate (MMF), activates *NRF2* signaling, upregulates the expression of antioxidant and anti-inflammatory response genes, increases mitochondrial membrane potential, and improves mitochondrial function, resulting in protective effects against CNS injury (8). DMF was approved by the FDA for the treatment of multiple sclerosis in 2013, and its application in the treatment of various tumors, inflammatory bowel disease, and intracranial hemorrhage has been reported in successive studies (9, 10). It has been demonstrated that treatment with DMF reduces the level of M1 macrophages and increases the level of M2 macrophages in the spleen and sciatic nerve of rats with autoimmune neuritis. Cellular experiments also confirmed that the shift in macrophage polarization from the M1 to M2 phenotype is dependent on DMF. In addition, DMF improved the inflammatory environment in the spleen of rats with autoimmune neuritis, characterized by the downregulation of messenger RNA (mRNA) levels of IFN-γ, TNF-α, IL-6, and IL-17 and upregulation of mRNA levels of IL-4 and IL-10 (11).

In this study, we have shown for the first time that DMF at a lower concentration inhibits the invasion of tumor cells by acting on TAMs. In further studies, DMF was found to increase the proportion of CD8^+^ T cells in tumors and inhibit the invasion of breast cancer. Mechanistically, we found that DMF reduced the release of reactive oxygen species (ROS) in macrophages by activating *NRF2*, thus weakening the ability of tumor cells for invasion. These results indicate that DMF plays an important role in the treatment of breast cancer.

## Material and Methods

### Cells

The 4T1 and RAW264.7 cells were purchased from Shanghai Institutes for Biological Sciences, Chinese Academy of Sciences. The 4T1 cells were cultured in RPMI 1640, RAW264.7 cells were cultured in Dulbecco’s Modified Eagle Medium supplemented with 10% fetal bovine serum (Gibco; Thermo Fisher Scientific, Waltham, MA, USA), 2 mM l-glutamine, 100 units/ml penicillin, and 100 units/ml streptomycin. The cells were maintained in a humidified atmosphere of 5% CO_2_ at 37°C.

### Reagent

DMF (purity>99%) was dissolved in 100% dimethyl sulfoxide (DMSO), and the final DMSO concentration in the cell culture did not exceed 0.1% throughout the study. Phycoerythrin (PE)-conjugated anti-mouse CD206 (Clone M1), PE-conjugated anti-mouse CCR7, PE-conjugated anti-mouse MGL1/2, PE-conjugated anti-mouse MHCII, and corresponding isotype controls were purchased from BD Pharmingen (San Diego, CA, USA). Penicillin, streptomycin, DCFH-DA, HRP-conjugated goat anti-mouse IgG (H+L), and MTT were purchased from Beyotime (Haimen, Jiangsu, China). The Annexin-V/PI apoptosis kit was purchased from BD Pharmingen. Matrigel and N-acetylcysteine (NAC) were purchased from Sigma-Aldrich (St. Louis, MO, USA).

### Macrophage/Tumor Cell Co-Culture

Co-culture experiments were performed according to the methods described by Wang et al. (12). A PET film 6-well hanging cell culture chamber (Millipore, Billerica, MA, USA) was used. RAW264.7 cells were co-cultured with 4T1 cells at a 1: 4 ratio in complete medium (CM) for 72 h. Serum concentrations of the two cell types were consistent.

### Phagocytosis Assays Using Latex Beads

Cells were washed with RPMI and then incubated with RPMI containing fluorescent red latex beads (1 μM diameter, L-2778; Sigma-Aldrich) for 2 h (number of microspheres: number of cells = 10:1). The cells were then washed three times with sterile pre-cooled phosphate-buffered saline (PBS) to wash down the uninternalized fluorescent microspheres. Analysis was performed using flow cytometry.

### Cell Invasion Assay

The cell invasion assay in this study was performed according to the methods described by He et al. (13). The ability of invasive cancer cells to migrate through Matrigel-coated filters was measured using transwell chambers (Costar, Cambridge, MA, USA) with polycarbonate membranes (8.0-μm pore size) coated with 100 μl of Matrigel (BD Biosciences, Franklin Lakes, NJ, USA) on the top side of the membrane. The upper surface of the matrix was challenged with 40,000 4T1 cells, and the cells were maintained in serum-free medium. The lower chamber contained a medium supplemented with 10% serum. After 24 h, the cells were stained with 0.1% crystal violet solution. Cells and Matrigel on the upper surface of the membrane were carefully removed using a cotton swab.

### Gene Expression Analysis

Total RNA was prepared from macrophages or tissues using TRIzol reagent (Invitrogen, Thermo Fisher Scientific). Total RNA (1.5 μg) was reverse transcribed using a first-strand cDNA synthesis kit (Bioteke, Beijing, China). Primers used in the real-time polymerase chain reaction (PCR) were GAPDH (mouse) (forward: 5’-AACTTTGGCATTGTGGAAGG-3’, reverse: 5’-ACACATTGGGGGTAGGAACA-3’), G6PD (mouse) (forward: 5’-CACAGTGGACGACATCCGAAA-3’, reverse: 5’-AGCTACATAGGAATTACGGGCAA-3’), IDH1 (mouse) (forward: 5’-ATGCAAGGAGATGAAATGACACG-3’, reverse: 5’-GCATCACGATTCTCTATGCCTAA-3’), GCLC (mouse) (forward: 5’-GGGGTGACGAGGTGGAGTA-3’, reverse: 5’-GTTGGGGTTTGTCCTCTCCC-3’), Txn1 (mouse) (forward: 5’-CATGCCGACCTTCCAGTTTTA-3’, reverse: 5’-TTTCCTTGTTAGCACCGGAGA-3’; PRDX1 (mouse) (forward: 5’-AATGCAAAAATTGGGTATCCTGC-3’, reverse: 5’-CGTGGGACACACAAAAGTAAAGT-3’). GAPDH was used as the reference gene. Quantitative PCR assays were carried out on a CFX96 real-time PCR detection system (Bio-Rad Laboratories, Hercules, CA, USA) using a qPCR kit (Bio-Rad Laboratories). The comparative threshold method for relative quantification was used, and the results were expressed as fold-change. The primers were synthesized by Invitrogen.

### Western Blotting

Cell proteins were extracted either through whole-cell lysis or using a nuclear protein extraction kit purchased from Beytotime (Haimen, Jiangsu, China), which contained protease and phosphatase inhibitors. The cell debris was removed by centrifugation at 4°C, and the supernatants were collected and stored at -70°C until use. The protein amounts were determined using the Pierce BCA protein assay kit (Thermo Fisher Scientific) according to the manufacturer’s instructions. For western blot analysis, the proteins were electrophoresed on a 10% sodium dodecyl sulfate-polyacrylamide gel electrophoresis, followed by immunoblotting on a polyvinylidene fluoride membrane (American Biosciences, Blauvelt, NY, USA). Immune complexes were incubated with peroxidase-labeled anti-rabbit or anti-mouse antibodies (Kangchen, Shanghai, China) for 2 h at room temperature. The blots were visualized using an enhanced chemiluminescent method kit (Sino-American Biotechnology, Henan, China). All uncropped data are shown in **Supplementary Figure 1**.

### Enzyme-Linked Immunosorbent Assay

At the end of co-culture, the transwell was removed and the concentrations of IL-1β, IL-6, IL-12, and IL-10 in the supernatant were assessed using enzyme-linked immunosorbent assay according to the manufacturer’s instructions.

### Flow Cytometry Analysis

For ROS analysis (14), production was examined using a DCFH-DA probe. After the stimulation medium was removed, cells were washed twice with 2 ml warmed PBS. Then, 1 mL PBS containing 10 μM DCFH-DA was added to the cells and incubated for 20 min at 37°C. The cells were then washed with PBS and subjected to flow cytometry analysis.

For apoptosis analysis (15), cells were stained with Annexin V-FITC in the presence of propidium iodide (PI) using an Annexin V-FITC apoptosis detection kit according to the manufacturer’s instructions (BD Biosciences).

For evaluation of macrophage phenotypes, RAW264.7 cells in different treatment conditions were incubated with PE-conjugated anti-mouse CD206 (Clone M1) antibody, PE-conjugated anti-mouse CCR7 antibody, PE-conjugated anti-mouse MGL1/2 antibody, and PE-conjugated anti-mouse MHCII antibody for 30 mins on ice followed by flow cytometry.

### Knockout of *NRF2* by CRISPR-CAS9

The gRNA (oligo 1: 5’- CACCGACTTGGAGTTGCCACCGCC-3’, oligo 2: 5’-AAACGGCGGTGGCAACTCCAAGTC-3’) designed to target the common exons of all mouse NRF-2 heterodimers was synthesized and cloned into lentiCRISPR v2 plasmid (Addgene 52961).

### Cell Cycle Analysis

The cell cycle distribution was analyzed using flow cytometry (16). The cells were immobilized overnight with 75% ethanol at 20°C and stained with 0.1% TritonX-100, 100 ng/ml PI, and 10 mg/ml RNase at 4°C for 30 mins. The proportion of cells in G1, S, and G2 phases was expressed using a DNA histogram.

### MTT Assay

An MTT assay was performed according to methods described in a previous study (17). Five thousand cancer cells were inoculated into 96-well plates and treated with different concentrations of DMF for 24 h and 48 h, with four replicates each, including untreated control groups. The MTT assay was used to detect adherent cells according to the manufacturer’s protocol (Beyotime). The average optical density of the control cells was 100% and the treatment results were expressed as a percentage of the control.

### *In Vivo* Assays

Six-week-old female BALB/c mice (Gempharmatech, Nanjing, China) were used for all animal experiments. All mice were housed in the Laboratory Animal Center at Southeast University. All protocols involving animal experiments were approved by the Ethics Committee of Southeast University (20200624007), and all methods were carried out following the relevant guidelines and regulations. When the cell concentration reached 80%, the cultured 4T1 cells were isolated from monolayer culture, washed with serum-free medium, and resuspended in RPMI 1640 (1×10^7^ cells/ml). Afterward, a 20 µl cell suspension was injected into the inguinal mammary fat pad of BALB/c mice with syngeneic immunological activity. The mice were euthanized by CO_2_ asphyxiation, and the tumors were collected for experimental assays.

### Preparation of Tumor Single-Cell Suspensions

The resected tumor tissue was cut into pieces and digested in RPMI-1640 medium containing 0.5 mg/mL collagenase I, 0.5 mg/mL collagenase IV, and 100 μg/mL Dnase I. After 1 h, the tumors were passed through a 40-μm cell strainer to remove the undigested tissue pieces. Erythrocyte lysis buffer was then added to the cells to remove erythrocytes, followed by washing the cells twice with PBS containing 2 mM EDTA to obtain single cell suspension. Single cell suspensions were blocked with mouse Fc receptor blocking reagent for 15 min at 4°C and then stained with antibody (1:100) for 1 h on ice.

### Statistical Analysis

Data are expressed as the mean ± standard error. Statistical analysis was performed using the Student’s *t*-test when only two value sets were compared. One-way ANOVA followed by Dunnett’s test was used when the data involved three or more groups. *P* < 0.05, *P* < 0.01, *P* < 0.001, and *P* < 0.0001 were considered statistically significant and are indicated by *, **, ***, or ****, respectively.

### Availability of Data and Materials

õThe datasets used and/or analyzed during the current study are available from the corresponding author upon reasonable request.

## Results

### DMF Inhibited the Pro-Invasion Ability of TAMs

**Figure 1:**
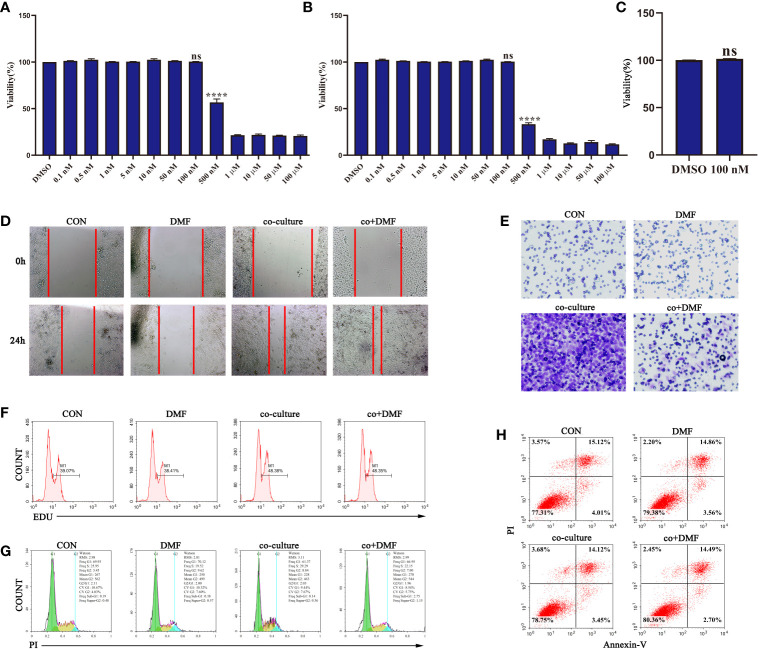
Dimethyl formamide (DMF) inhibits the pro-invasion ability of tumor-associated macrophages (TAMs). **(A)** The viability of RAW264.7 cells was detected by MTT assay after treatment with different concentrations of DMF for 24 h. **(B)** The viability of RAW264.7 cells was detected by MTT assay after treatment with different concentrations of DMF for 48 h. **(C)** The viability of 4T1 tumor cells was evaluated by MTT assay after treatment with 100 nM DMF for 48 h. **(D–H)** In the presence or absence of DMF, 4T1 tumor cells were cultured alone or co-cultured with RAW264.7 cells for 48 h. Scratch migration assay **(D)**, invasion assay **(E)**, proliferation assay **(F)**, cycle analysis **(G)**, and apoptosis analysis **(H)** were performed. Data are presented as the mean ± standard error and represent at least three independent experiments with three replicates. ns, no significance; ****P < 0.0001 as determined by the one-way ANOVA test.

First, we analyzed the effect of DMF on the viability of RAW264.7 macrophages at different concentrations using the MMT assay. The results showed that DMF had a significant inhibitory effect on the viability of RAW264.7 macrophages when the concentration was higher than 100 nM (**Figures 1A, B**). Additionally, 100 nM DMF did not affect the viability of 4T1 tumor cells (**Figure 1C**). Therefore, 100 nM DMF is a suitable dose for culturing cell models *in vitro*. Furthermore, we constructed a co-culture system of macrophages and tumor cells *in vitro* to induce the differentiation of macrophages into TAM. In the co-culture system, the presence of macrophages significantly enhanced the invasion and migration abilities of the 4T1 tumor cells (**Figures 1D, E**). DMF did not affect the TAM pro-migration ability (**Figure 1D**), but significantly inhibited TAM pro-invasion ability (**Figure 1E**). We also examined the effect of DMF on the proliferation, apoptosis, and cell cycle of 4T1 cells in the co-culture system (**Figures 1F–H**). There was no difference in proliferation, apoptosis, and cell cycle. The results showed that DMF specifically affected the invasion-promoting ability of TAMs.

### DMF Inhibited the Pro-Invasion Ability of TAMs by Reducing ROS Production

**Figure 2:**
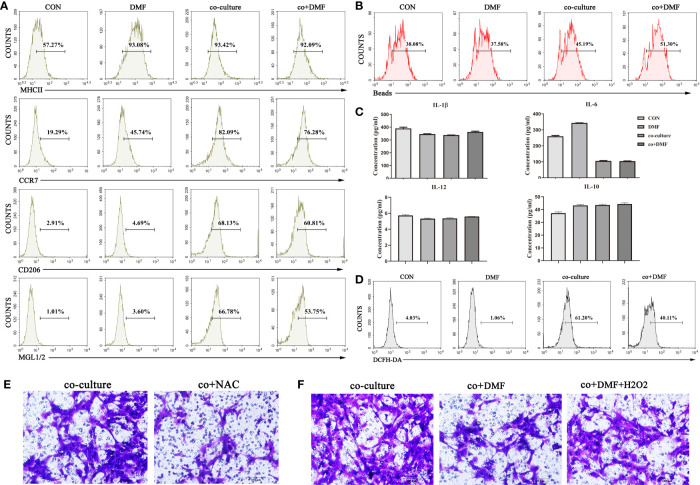
DMF inhibits the pro-invasion ability of TAMs by reducing reactive oxygen species (ROS) production. **(A)** In the presence or absence of DMF, RAW264.7 cells were cultured alone or co-cultured with 4T1 cells for 48 hours, and the expression of membrane surface molecules (MHCII, CCR7, CD206, MGL1/2) of RAW264.7 cells was detected by flow cytometry. **(B)** In the presence or absence of DMF, RAW264.7 cells were cultured alone or co-cultured with 4T1 cells for 48 hours, and the phagocytosis of RAW264.7 cells was detected by flow cytometry. **(C)** In the presence or absence of DMF, RAW264.7 cells were cultured alone or co-cultured with 4T1 cells for 48 hours, and the secretion levels of TNFα, IL-6, IL-12, and IL-10 of RAW264.7 cells were assessed by enzyme-linked immunosorbent assay. **(D)** In the presence or absence of DMF, RAW264.7 cells were cultured alone or co-cultured with 4T1 cells for 48 hours, and ROS production in RAW264.7 cells was detected by flow cytometry. **(E)** In the presence or absence of N-acetylcysteine, 4T1 cells and RAW264.7 cells were co-cultured for 48 hours to detect the invasion ability of 4T1. **(F)** In the presence or absence of DMF or H_2_O_2_, 4T1 cells were co-cultured with RAW264.7 cells for 48 hours to assess the invasion ability of 4T1 cells. Data are presented as the mean ± SEM and represent at least three independent experiments with three replicates.

To assess the effect of DMF on macrophage phenotype, flow cytometry was used to analyze the expression of membrane molecules (MHCII, CCR7, CD206, MGL1/2) in macrophages after co-culture. We found that DMF did not affect the expression of macrophage membrane molecules (**Figure 2A**). At the same time, a phagocytosis assay using fluorescent red latex beads showed that DMF did not affect phagocytosis (**Figure 2B**) of macrophages. Furthermore, we evaluated the levels of the inflammatory cytokines in the co-culture system in the presence of DMF. The results showed that there was no difference in the secretion of IL-6, IL-10, IL-12, and TNFα (**Figure 2C**). ROS assay using a DCFH-DA probe showed that DMF decreased ROS production in macrophages (**Figure 2D**). ROS plays an important role in the initiation and progression of cancer (18) and stimulates tumor progression by promoting cell proliferation, survival, invasion, and metastasis (19). In the co-culture system, NAC, an antioxidant agent, inhibits ROS production. The results showed that the invasion ability of tumor cells was significantly inhibited (**Figure 2E**). Furthermore, we added H_2_O_2_ to simulate ROS while DMF was used. The results showed that the invasion-promoting ability of TAM increased (**Figure 2F**). In short, these results show that DMF inhibits TAM invasion by reducing ROS production.

### DMF Inhibited Tumor Growth and Metastasis in a 4T1 Orthotopic Inoculation Model of Breast Cancer

**Figure 3:**
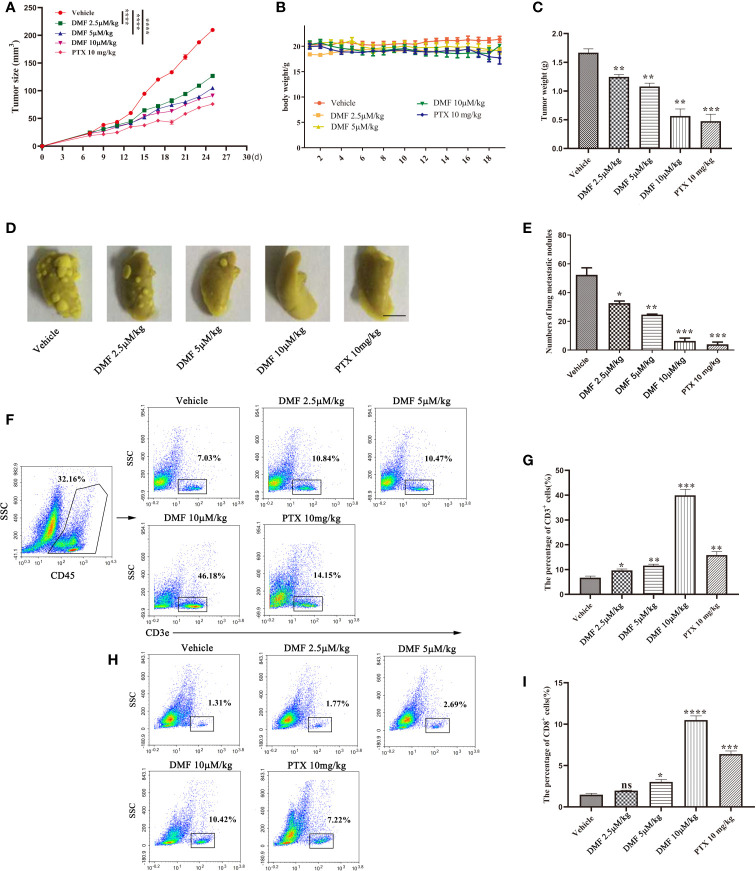
DMF inhibits tumor growth and metastasis in 4T1 orthotopic inoculation model of breast cancer. **(A)** Tumor growth of mice in each group was analyzed by measuring the volume every 2 days (n = 5). **(B)** Weight of each group of mice was measured once every two days (n = 5). **(C)** The tumor weight of each group of mice was analyzed (n = 5). **(D, E)** resection of lung tissue and counting of nodules to evaluate the anti-metastasis effect of oral DMF. Scale bar, 250 mm. **(F, G)** Flow cytometry analysis and quantification of CD3^+^ T cells in the tumor microenvironment of each group. **(H, I)** Flow cytometry analysis and quantification of CD8^+^ T cells in the tumor microenvironment of each group. Data are presented as the mean ± SEM. ns, no significance. *P < 0.05, **P < 0.01, ***P < 0.001, ****P < 0.0001 as determined by the one-way ANOVA test.

To further evaluate the effect of DMF on tumors *in vivo*, we constructed a 4T1 orthotopic inoculation model of breast cancer. One week after 4T1 cells were injected into the inguinal mammary fat pads of BALB/c mice, DMF with different concentrations (2.5 µ M/kg, 5 µM/kg, or 10 µM/kg) or vehicle was administered intragastrically once every 2 days (n=5), and paclitaxel (a chemotherapeutic drug for breast cancer in clinical treatment) was used as a positive control. The experiment was terminated on the 28th day. To evaluate the effect of DMF on tumor growth and progression, the tumor size (length, width) was measured using a caliper, and the tumor volume was calculated according to the formula (length × width^2^)/2. As shown in **Figures 3A, C**, DMF significantly inhibited the growth of 4T1 tumors compared to control, but there was no significant difference in body weight (**Figure 3B**). In addition, we also observed that the lung metastasis of 4T1 tumors was significantly inhibited by DMF (**Figures 3D, E**). Furthermore, we analyzed the infiltration of T cells into the tumor microenvironment. The results showed that after DMF administration, T cell infiltration was significantly upregulated (**Figures 3F–I**). These data demonstrate the *in vivo* therapeutic effects of DMF on breast cancer.

### DMF Inhibits Tumor Metastasis in MMTV-PyMT Spontaneous Breast Cancer Model

**Figure 4:**
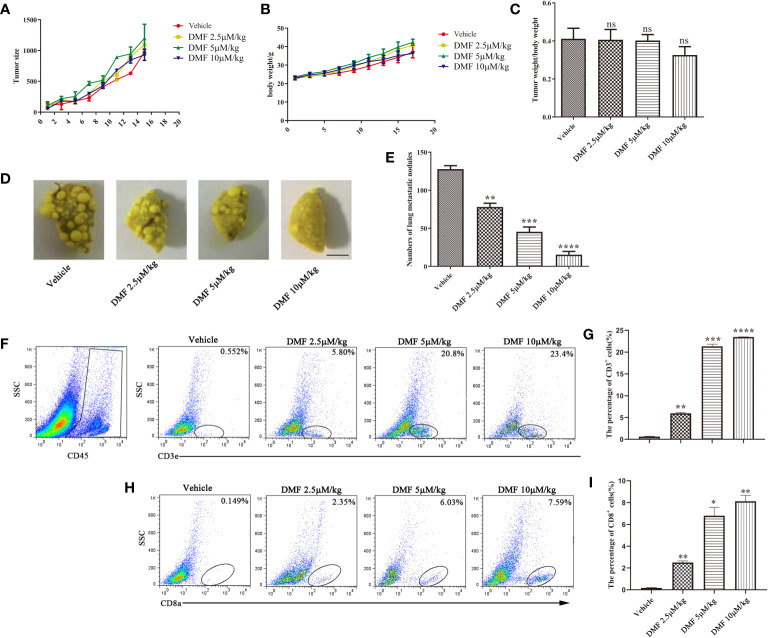
DMF inhibits tumor metastasis in MMTV spontaneous breast cancer model. **(A)** Tumor growth of mice in each group was analyzed by measuring volume every 2 days (n = 5); **(B)** Weight of each group of mice is measure once every two days (n = 5). **(C)** The tumor weight of each group of mice was analyzed (n = 5). **(D, E)**, resection of lung tissue and counting of nodules to evaluate the anti-metastasis effect of oral DMF; Scale bar, 250 mm. **(F, G)** Flow cytometry analysis and quantification of CD3^+^ T cells in the tumor microenvironment of each group. **(H, I)** Flow cytometry analysis and quantification of CD8^+^ T cells in the tumor microenvironment of each group. Data are presented as the mean ± SEM. ns, no significance. *P < 0.05; **P < 0.01; ***P < 0.001; ****P < 0.0001 as determined by the one-way ANOVA test.

MMTV-PyMT transgenic mice are a type of spontaneous breast cancer model (20). From the perspective of tumor occurrence, the model is remarkably similar to human breast cancer, and the experimental results are beneficial in serving as the basis for future clinical research. Therefore, we evaluated the efficacy of DMF in this model. The administration process was consistent with that of the 4T1 orthotopic inoculation model. The results showed that although DMF had little effect on tumor weight (**Figures 4A–C**), lung staining showed that DMF significantly inhibited tumor lung metastasis (**Figures 4D, E**). Furthermore, we observed an increase in CD8^+^ T cell infiltration in the tumor microenvironment from DMF-treated mice (**Figures 4F–I**), but there was no difference in tumor size, indicating that the increase in CD8^+^ T cells was not sufficient to inhibit tumor growth.

### DMF Inhibits TAM Pro-Invasion Ability *via* the Activation of *NRF2*

**Figure 5:**
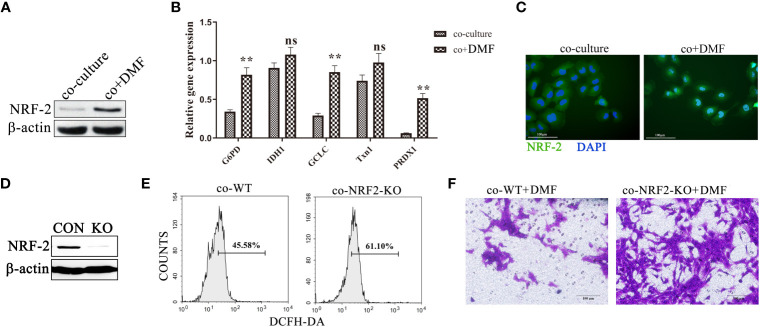
DMF inhibits TAM pro-invasion ability *via the* activation of *NRF2*. **(A, B)** In the presence or absence of DMF, RAW264.7 and 4T1 cells were co-cultured for 48 h. Western blotting was used to detect the expression of *NRF2* in RAW264.7, and qPCR was used to detect the mRNA levels of G6PD, IDH1, GCLC, Txn1, and PRDX1 in RAW264.7 cells. **(C)** Representative fluorescent images of NRF2 localization in RAW264.7 cells co-cultured with 4T1 cells in the presence or absence of DMF. **(D)** Western blot analysis of the expression of NRF-2 in RAW264.7 cells with and without NRF-2 knockout. **(E)** Control RAW264.7 and *NRF2* knockout RAW264.7 cells were cultured with 4T1 tumor cells for 48 h. ROS production was detected by flow cytometry. **(F)** In the presence of DMF, 4T1 tumor cells were co-cultured with control RAW264.7 and *NRF2* knockout RAW264.7 cells for 48 h to detect their invasion ability. Data are presented as the mean ± standard error and represent at least three independent experiments with three replicates. ns, no significance; **P < 0.01, as determined by Student’s *t*-test.

DMF is a pharmacological activator of *NRF2*. We speculated whether DMF activated NRF2 in macrophages in the co-culture system. Western blotting showed that the expression of *NRF2* in co-cultured macrophages increased after DMF stimulation (**Figure 5A**), and qPCR showed that the expression of genes downstream of *NRF2* was significantly upregulated (**Figures 5B, C**). Additionally, we observed that 100 nM DMF did not activate the *NRF2* pathway and reduced ROS production in 4T1 tumor cells (**Supplementary Figures 2A–C**). We constructed *NRF2* knockout macrophages (**Figure 5D**) and found that *NRF2* knockout significantly enhanced ROS production after co-culture with tumor cells in the presence of DMF (**Figure 5E**). DMF did not inhibit the ability to pro-invasion ability of TAMs (**Figure 5F**), indicating that DMF weakened the ability of TAMs to promote tumor invasion by activating *NRF2*.

## Discussion

In this study, we demonstrated for the first time that DMF significantly reduces the pro-invasion ability of TAMs and inhibits tumor metastasis in 4T1 orthotopic breast cancer and MMTV spontaneous breast cancer models. Although the direct cytotoxic effect of DMF on tumor cells has been reported (21), immune function is very important for DMF to inhibit tumor growth since the SCID mice injection model which lacks functional lymphocytes does not respond to DMF treatment. In addition, Nrf2 is a key regulator of myeloid-derived suppressor cells (MDSCs), suggesting that innate immune cells may be an additional target of the *NRF2* agonist DMF (22).

Although DMF has a strong anti-inflammatory activity, it also has toxic adverse effects that would warrant minimizing the therapeutic concentration of DMF. It has been shown that only low concentrations of DMF treatment are effective in reducing disease severity in chronic sponge-skin autoimmune mice compared to high concentrations (23). In our research, we chose a suitable concentration of DMF, which is non-toxic to tumor cells and macrophages. At this lower concentration, it effectively inhibited the pro-invasion ability of TAMs.

The tumor immunosuppressive microenvironment does not only promote the development of tumors but is also the main obstacle to effective tumor immunotherapy. TAMs are the dominant myeloid cell group in breast cancer and are the main source of immunosuppression. Altering the activation of TAMs may overcome this obstacle. We found that DMF not only inhibits TAM pro-invasion ability but also increases T cell infiltration in the tumor microenvironment to improve the tumor immune microenvironment, which indicates that DMF combined with other immunotherapy strategies (e.g., immune checkpoint blocking, immune activator, etc.) may eliminate cancer cells more effectively with minimal side effects.

Tumor cell invasion, angiogenesis, and metastasis are interrelated processes that represent the final and most destructive malignant stage. This process includes cell growth, proliferation, and migration. Evidence accumulated from *in vitro* and *in vivo* studies in the past few years showed that ROS is the signal for angiogenesis and metastasis (24, 25). ROS have been shown to mediate these effects by inducing transcription factors and genes to participate in angiogenesis and metastasis. However, the role of ROS in regulating tumor cell metastasis and angiogenesis seems to be contradictory. A high level of ROS inhibits tumor formation and metastasis by destroying cancer cells while the suboptimal concentration encourages cancer cell metastasis (26). ROS also has the potential to promote angiogenesis and metastasis of tumor cells in animal models of breast cancer, bladder cancer, lung cancer, melanoma, sarcoma, colon cancer, and prostate cancer. Catalase can significantly reduce the invasive behavior of tumor cells in a transgenic mouse model of metastatic breast cancer (MMTV-PyMT) (27). In a mouse bladder cancer model, ROS-induced metastasis by stimulating NF-κB has also been shown to be caused by ROS production and upregulation of NF-κB and MMP-9 in a mouse model (28). It is worth noting that in the mouse melanoma model, surgical methods for tumor resection have been proven to induce ROS production and promote the growth of metastatic tumors. Similarly, our research shows that H_2_O_2_ increases the invasion ability of tumor cells, while DMF effectively reduces ROS production in TAM, thus weakening the invasion ability of tumor cells.

In summary, our results showed that DMF, an antioxidant, reduces ROS production in TAMs at low concentrations, which leads to a lower invasion ability of cancer cells. Therefore, reducing the production of ROS as a cancer treatment may be a viable approach, and DMF may be a promising anticancer agent.

## Data Availability Statement

The original contributions presented in the study are included in the article/**Supplementary Material**. Further inquiries can be directed to the corresponding author.

## Ethics Statement

The animal study was reviewed and approved by The Ethics Committee of Southeast University.

## Author Contributions

YL designed the experiments, and YJ and YX performed the experiments. YL and YJ wrote the main manuscript text, and YX prepared **Figures 1** **–** **5**. All authors contributed to the article and approved the submitted version.

## Conflict of Interest

The authors declare that the research was conducted in the absence of any commercial or financial relationships that could be construed as a potential conflict of interest.

## Publisher’s Note

All claims expressed in this article are solely those of the authors and do not necessarily represent those of their affiliated organizations, or those of the publisher, the editors and the reviewers. Any product that may be evaluated in this article, or claim that may be made by its manufacturer, is not guaranteed or endorsed by the publisher.

## Abbreviations

DMF, Dimethyl Formamide; ROS, Reactive Oxygen Species; TAM, Tumor-associated Macrophages; MMTV-PyMT, Murine Mammary Tumor Virus- Polyoma Middle T Antigen; CON, Control.
