# Supplementary material for: DMF Activates NRF2 to Inhibit the Pro-Invasion Ability of TAMs in Breast Cancer
Source: Front Oncol. 2021 Aug 12;11:706448. doi: 10.3389/fonc.2021.706448 (PMC8406629; doi:10.3389/fonc.2021.706448)
Supplement: Supplementary file 1 [file DataSheet_1.pdf]

DMF activates *NRF2* to inhibit the pro-invasion ability of TAMs in breast cancer

Ying Li[1,2], Yaxu Jia[1], Yurong Xu[1], Kan Li[1*]

1. Department of Clinical Laboratory, Affiliated Zhongda Hospital of Southeast University, Nanjing, China
2. Department of Epidemiology, School of Public Health of Suzhou University, Suzhou, China

* Correspondence: likan15@163.com

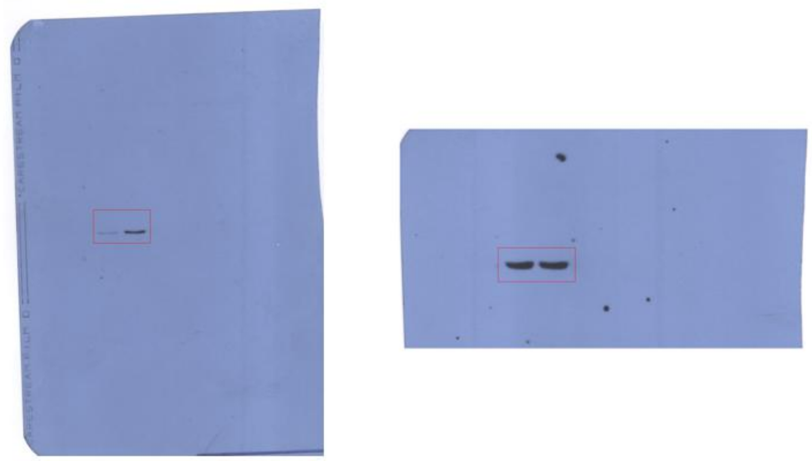

Figure5A

Supplementary Fig.1. Uncropped data shown.

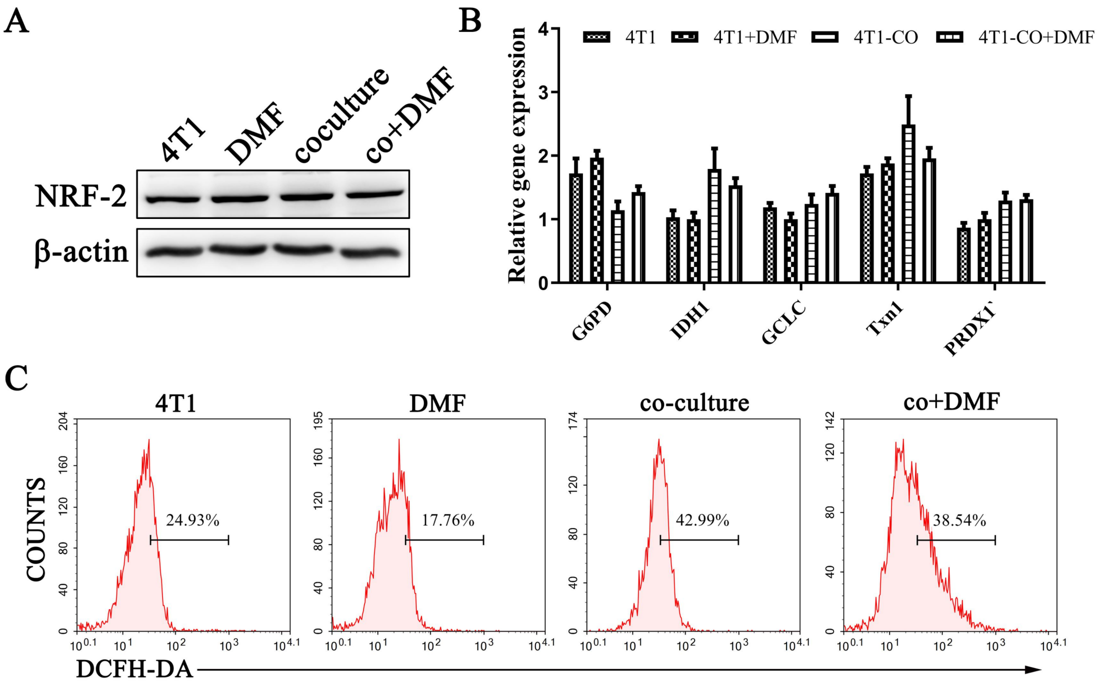

Supplementary Fig.2. 100 nM DMF did not activate the Nrf-2 pathway and reduce the ROS production.

A-C, in the presence or absence of DMF, 4T1 tumor cells cultured alone or cocultured with

RAW264.7 cells for 48 hours. Western was used to detect the expression of Nrf2 in 4T1 tumor cells. q-PCR was used to detect the mRNA levels of G6PD, IDH1, GCLC, Txn1 and PRDX1 in 4T1 tumor cells, and ROS production was detected by flow cytometry.
